# ﻿The ECAT dataset: expert-validated distribution data of endemic and sub-endemic trees of Central Africa (Dem. Rep. Congo, Rwanda, Burundi)

**DOI:** 10.3897/phytokeys.206.77379

**Published:** 2022-09-16

**Authors:** Wesley Tack, Henry Engledow, Nuno Veríssimo Pereira, Christian Amani, Steven P. Bachman, Patricia Barberá, Henk J. Beentje, Gaël U. D. Bouka, Martin Cheek, Ariane Cosiaux, Gilles Dauby, Petra De Block, Corneille E. N. Ewango, Eberhard Fischer, Roy E. Gereau, Serene Hargreaves, Yvette Harvey-Brown, Davy U. Ikabanga, Edouard Ilunga wa Ilunga, James Kalema, Peris Kamau, Olivier Lachenaud, Quentin Luke, Ithe Mwanga Mwanga, Sydney T. Ndolo Ebika, Jacques Nkengurutse, Aimable Nsanzurwimo, Salvator Ntore, Sophie L. Richards, Reddy Shutsha Ehata, Murielle Simo-Droissart, Tariq Stévart, Marc S. M. Sosef

**Affiliations:** 1 Meise Botanic Garden, Nieuwelaan 38, 1860 Meise, Belgium Meise Botanic Garden Meise Belgium; 2 Université Officielle de Bukavu, Bukavu, Democratic Republic of the Congo IUCN SSC Eastern African Plant Red List Authority (EAPRLA) Gland Switzerland; 3 IUCN SSC Eastern African Plant Red List Authority (EAPRLA), Gland, Switzerland Université Officielle de Bukavu Bukavu Democratic Republic of the Congo; 4 Royal Botanic Gardens, Kew, Richmond, Surrey, UK Royal Botanic Gardens Richmond United Kingdom; 5 Missouri Botanical Garden, Africa & Madagascar Department, St. Louis, MO 63110, USA Missouri Botanical Garden, Africa & Madagascar Department St. Louis United States of America; 6 Laboratoire de Biodiversité, de Gestion des Ecosystèmes et de l’Environnement, Faculté des Sciences et Techniques, Université Marien Ngouabi, BP 69, Brazzaville, Democratic Republic of the Congo Université Marien Ngouabi Brazzaville Democratic Republic of the Congo; 7 Plant Systematics and Ecology Laboratory, University of Yaoundé I, P.O. Box 047, Yaoundé, Cameroon University of Yaoundé I Yaoundé Cameroon; 8 Institut de Recherche pour le Développement, Université de Montpellier, Montpellier, France Université de Montpellier Montpellier France; 9 AMAP, Université de Montpellier, CIRAD, CNRS, INRAE, IRD, Montpellier, France Université de Kisangani Kisangani Democratic Republic of the Congo; 10 Centre de Surveillance de la Biodiversité, Université de Kisangani, Kisangani, Democratic Republic of the Congo University of Koblenz and Landau Koblenz Germany; 11 University of Koblenz-Landau, Universitätsstraße 1, Koblenz, 56070, Germany IUCN SSC Central Africa Plant Red List Authority (CARLA) Gland Switzerland; 12 IUCN SSC Central Africa Plant Red List Authority (CARLA), Gland, Switzerland Botanic Gardens Conservation International Richmond United Kingdom; 13 Botanic Gardens Conservation International, Richmond, Surrey, UK University of Sciences and Techniques of Masuku Franceville Gabon; 14 Department of Biology, Faculty of Sciences, University of Sciences and Techniques of Masuku, BP: 941, Franceville, Gabon Université de Lubumbashi Lubumbashi Democratic Republic of the Congo; 15 Herbarium de Lubumbashi, Université de Lubumbashi, 1825, Route Kasapa, Lubumbashi, Democratic Republic of the Congo Makerere University Herbarium, Department of Plant Sciences Microbiology and Biotechnology Kampala Uganda; 16 Makerere University Herbarium, Department of Plant Sciences Microbiology and Biotechnology, P.O. Box 7062, Kampala, Uganda East African Herbarium, National Museums of Kenya Nairobi Kenya; 17 East African Herbarium, National Museums of Kenya, P.O. Box 45166-00100, Nairobi, Kenya Université Libre de Bruxelles Brussels Belgium; 18 Herbarium et Bibliothèque de Botanique africaine, C.P. 265, Université Libre de Bruxelles, Campus de la Plaine, Boulevard du Triomphe 1050, Brussels, Belgium Centre de Recherche en Sciences Naturelles CRSN/Lwiro, Laboratoire de Systématiquement et Taxonomie végétale Bukavu Democratic Republic of the Congo; 19 Centre de Recherche en Sciences Naturelles CRSN/Lwiro, Laboratoire de Systématiquement et Taxonomie végétale, D.S. Bukavu, Democratic Republic of the Congo University of Burundi Bujumbura Burundi; 20 Department of Biology, Faculty of Science, University of Burundi, P.O. Box 2700, Bujumbura, Burundi Department of Biotechnologies, Faculty of Applied Sciences, INES-Ruhengeri Ruhengeri Rwanda; 21 Department of Biotechnologies, Faculty of Applied Sciences, INES-Ruhengeri, B.P.155 Ruhengeri, Rwanda UCN SSC Central Africa Plant Red List Authority (CARLA) Gland Swaziland

**Keywords:** Africa, conservation, data capture, data cleaning, endemics, flora, flowering plants, geographic range, herbarium, IUCN Red List, threatened

## Abstract

In this data paper, we present a specimen-based occurrence dataset compiled in the framework of the Conservation of Endemic Central African Trees (ECAT) project with the aim of producing global conservation assessments for the IUCN Red List. The project targets all tree species endemic or sub-endemic to the Central African region comprising the Democratic Republic of the Congo (DR Congo), Rwanda, and Burundi. The dataset contains 6361 plant collection records with occurrences of 8910 specimens from 337 taxa belonging to 153 genera in 52 families. Many of these tree taxa have restricted geographic ranges and are only known from a small number of herbarium specimens. As assessments for such taxa can be compromised by inadequate data, we transcribed and geo-referenced specimen label information to obtain a more accurate and complete locality dataset. All specimen data were manually cleaned and verified by botanical experts, resulting in improved data quality and consistency.

## ﻿Introduction

The alarming rate of biodiversity loss worldwide has increased the need to conduct conservation assessments for the International Union for Conservation of Nature (IUCN) Red List of Threatened Species (Rodrigues et al. 2006; Mace et al. 2008). In particular, plants, especially those from species-rich tropical forests, need to be better represented on the IUCN Red List to obtain a representative picture of the state of biodiversity as a whole and to help inform natural resource management and conservation planning (Collen et al. 2008; Stuart et al. 2010). Africa’s rainforests, including most in DR Congo, are particularly threatened by climate change and rapid population growth (Réjou-Méchain et al. 2021; Sosef et al. 2021), but a lack of data hampers the preparation of extinction risk assessments (Stévart et al. 2019).

The IUCN Red List assessment procedure uses numerical thresholds within five criteria to classify taxa according to their relative risk of extinction. Criterion B (restricted geographic range) is the most frequently used for plants (Collen et al. 2008; Le Breton et al. 2019), mainly because it does not require population demographic data, which are rarely available for plants and especially for tropical species. Instead, criterion B allows taxa to be classified as threatened when their geographic range, measured as either the extent of occurrence (EOO) or the area of occupancy (AOO), falls below certain thresholds, provided that at least two out of three additional subcriteria are met concerning its population: (a) severely fragmented or known to exist in no more than a given number of threat-defined locations; (b) continuing decline; or (c) extreme fluctuations (IUCN Standards and Petitions Committee 2019).

The first and perhaps most time-consuming task in preparing conservation assessments under criterion B is to obtain a realistic view of a taxon’s past and current distribution. For tropical plants, this is generally derived from herbarium specimens. Although large-scale digitisation programmes have increased the availability of digital biodiversity data, the specimen data are far from complete, up-to-date, accurate, or clean (Graham et al. 2004; Yesson et al. 2007; Nelson and Ellis 2018; Zizka et al. 2020b). On the contrary, limitations in the quantity and quality of plant occurrence data along taxonomic, geographic, and temporal dimensions may hamper their use in research and conservation applications (Meyer et al. 2016). Many specimens in herbaria and databases lack geographic coordinates (Nic Lughadha et al. 2018), requiring additional geo-referencing before calculating range statistics. This is especially relevant for uncommon taxa known from only a few collections, where the inclusion of extra geo-referenced specimen data may cause some parameters used in the assessment process to exceed one of the thresholds, thereby changing the conservation status (Miller et al. 2012). Improving data quality is equally important to obtain the most complete and accurate locality data. Before any spatiotemporal inferences can be drawn from herbarium collections, numerous issues need to be addressed, including conflicting taxonomy, synonymy, misidentifications, imprecise or erroneous coordinates, and duplicate specimens (Soberón and Peterson 2004; Nic Lughadha et al. 2018). As the EOO is affected by geographic outliers, taxonomic and spatial errors could lead to a miscalculation of the extinction risk, especially for threatened taxa (Panter et al. 2020). Several tools and workflows have been developed to implement automated data cleaning, such as Biogeo (Robertson et al. 2016), SpeciesGeoCoder (Töpel et al. 2017), CoordinateCleaner (Zizka et al. 2019), and BDcleaner (Jin and Yang 2020), to mention a few. These tools have proven valuable in detecting and flagging suspect data records that require further inspection but do not provide mechanisms to resolve these issues efficiently. Users may decide to remove suspect records, but this may affect downstream analyses (Maldonado et al. 2015; Zizka et al. 2020a). In our situation, working with poorly-sampled and often range-restricted endemic taxa, correcting such errors is a vital step in ensuring that the assessments use the best available evidence, as advocated by the IUCN (IUCN Standards and Petitions Committee 2019). Also, conservation assessments should be preferably carried out on manually cleaned and expert-validated data (Hjarding et al. 2015; Panter et al. 2020).

Here, we provide a high-quality, expert-validated occurrence dataset compiled by the ECAT project, which is part of the larger Global Tree Assessment (GTA) coordinated by Botanic Gardens Conservation International (BGCI 2021). The ECAT project aimed to prepare global conservation assessments for all trees endemic or sub-endemic to the region comprising DR Congo, Rwanda, and Burundi, hereafter referred to as Central Africa. It was executed in collaboration with the IUCN SSC Central African and Eastern African Plant Red List Authorities (CARLA and EAPRLA). We summarise how the data was compiled, which included normalisation, harmonisation, aggregation, data transcription, geo-referencing, quality control, data cleaning, and validation by data managers and botanical experts. We conclude by highlighting the taxonomic, spatial, and temporal coverage of the data. The ECAT dataset was not only used to develop or update the conservation assessment of 347 Central African tree taxa, many of which are threatened with extinction, but will also be used in a series of future studies (e.g., studying the effectiveness of the protected area network in the conservation of threatened tree species). Through this project, we hope to support and help guide effective management and conservation strategies to preserve the unique plant diversity of Central Africa.

## ﻿Project details

### ﻿Project title

Conservation of Endemic Central African Trees (ECAT) through IUCN Red Listing and Species Distribution Modelling.

### ﻿Funding

Funding for the ECAT project was provided by the Franklinia Foundation, with a substantial in-kind contribution from Meise Botanic Garden and Missouri Botanical Garden.

### ﻿Study area

Central Africa, as defined in this study, covers a total of 2.4 million square km, comprising the countries of DR Congo, Rwanda, and Burundi and stretching from a narrow coastal strip at the western border of DR Congo (excluding the Cabinda enclave) to the montane region of the Albertine Rift. The core of this region consists of the Congo Basin, which is the second largest tropical forest area in the world after the Amazon Basin, with much of the area being at low elevation (below 600 m). The natural vegetation of the Congo Basin is classified as Guineo-Congolian rainforest on well-drained sites, with swamp forest on hydromorphic soils (White 1983). In the south-east of DR Congo, at an elevation ranging from ca. 600 m at Lake Upemba to 1750 m in the Hauts-Plateaux, the Zambezian forest-savannah mosaic is dominated by dry tropical woodland (miombo) interspersed with savannah and remnants of dry evergreen woodland (muhulu) (Meerts and Hasson 2016; Pierre Meerts, pers. comm.). Similar dry forests can be found in the northern region of DR Congo, bordering the Central African Republic and South Sudan, forming part of the Guineo-Sudanian phytoregion (White 1983; Droissart et al. 2018). The Central African region is home to an estimated 11,000 vascular plant species (Sosef 2016), of which more than 1800 are endemic (Sosef et al. 2017).

## ﻿Methods

Based on data available at Meise Botanic Garden, supplemented with data from the BGCI GlobalTreeSearch (Beech et al. 2017), we compiled a list of 347 tree taxa endemic or sub-endemic to Central Africa, including their geographic distribution and nomenclatural synonyms (totalling 481 names). Accepted names generally follow the African Plant Database (version 3.4.0. 2018), which for Central African taxa draws heavily on the Flore d’Afrique centrale, Flora of Tropical East Africa, and Flora Zambesiaca. We defined a tree as any woody single-stemmed plant at least 3 m tall. Taxa still met this definition if they usually occur as a shrub or a liana and only occasionally in the form of a tree. This list was then verified by the IUCN SSC Global Tree Specialist Group.

We differentiated between endemic and sub-endemic taxa based on their spatial distribution relative to the land borders of DR Congo, Rwanda, and Burundi. We considered 219 taxa as Central African true endemics as their current distribution range is restricted to DR Congo (186 taxa), Rwanda (3), Burundi (2), or a combination of these three countries (28). The remaining 128 taxa from our list were deemed sub-endemic to Central Africa. For 116 of these sub-endemic taxa, all herbarium specimens in our dataset originated from the area delineating DR Congo, Rwanda, and Burundi, extended by a 5-degree buffer zone. For the remaining 12 sub-endemic taxa in our study, most specimens were from Central Africa (70–94%), with only a few collected outside the 5-degree buffer zone (1–23%).

We retrieved the specimen data for these taxa and their synonyms from our institutional collection database (BR; all herbarium acronyms according to Thiers 2018) and supplemented them with data from RAINBIO, a database of tropical African vascular plants distributions (Dauby et al. 2016). RAINBIO contains geo-referenced specimen records from a number of institutional collections with a strong focus on Africa, together with several personal databases collated by individual researchers (for details on the construction of the RAINBIO database and the quality checks performed, see Dauby et al. 2016). Occasionally, missing specimen records were added from online institutional data portals such as TROPICOS and JSTOR-Global Plants, while some verified specimen records were added from other sources such as taxonomic revisions and floras (e.g., Flora of Tropical East Africa, Flora Zambesiaca). The specimen data from all these different sources were thoroughly pre-processed before aggregating them into one comprehensive dataset. For instance, discrepancies in taxon names were resolved and harmonised with respect to synonymy and re-identifications, two-digit country codes were employed in accordance with ISO 3166, collector names were standardised, and collecting dates were converted to YYYY-MM-DD format. The dataset was meticulously reviewed and edited to merge duplicate specimen records (specimens of the same taxon made by the same collector at the same place and time, usually collected from the same tree). These duplicates often did not have the same quantity and quality of transcribed metadata due to missing information on some specimen labels, incomplete label transcription in the database, or transcription errors. When merging duplicates, we took care to retain all metadata relevant to Red List assessors and to resolve any inconsistencies.

Transcription of specimen labels is often restricted to selected data fields due to resource constraints. As a result, a considerable amount of descriptive information relevant to Red List assessments may be missing from specimen databases. To enrich our data, we transcribed specimen label data focusing mainly on gaps in the locality description, habitat, and elevation. The newly transcribed data allowed us to geo-reference several specimens without coordinates and improve the geo-referencing accuracy of others. Although recent herbarium specimens increasingly contain accurate coordinates captured in real-time using a GPS device, this is not the case for the bulk of the Central African collection at BR that predated GPS devices. It was often possible to infer the geographic coordinates from the transcribed data using historical topographic maps and gazetteers or by checking the collector’s itinerary. Specimen records that could not be geo-referenced because the locality description was missing, too vague, or unclear (e.g., illegible handwriting) were removed from the dataset.

The dataset was checked for any spatial errors through an iterative series of inspections. First, we used the R package CoordinateCleaner version 2.0–18 (Zizka et al. 2019) to flag records with potentially erroneous coordinates, including those that fall in the ocean or outside the indicated country, those that coincide with country and province centroids, and those with zero latitudes or longitudes. Next, we checked the data for intrinsic consistency in a GIS environment to flag additional problematic records. For example, we verified that the coordinates fell within the province or district as stated on the specimen label and checked the collector’s itinerary to evaluate the geo-referencing quality in suspect cases. This approach revealed several spatial errors missed by the automated cleaning. We checked all flagged records one by one and made corrections where appropriate. Common causes of spatial errors were the inversion of latitude and longitude, the lack of a minus sign for south or west, coordinate transformation errors, and typographical errors. Even after extensive data cleaning, some geographic discrepancies remained. Occurrences falling in a country different from the one stated on the specimen label but for which the distance to the land border was less than 5 km were retained in our dataset. We considered this margin acceptable given the uncertainties associated with natural history data, the precision of the locality data, and the spatial precision of the country GIS layers used. Other records whose coordinates were considered incorrect or too imprecise for our purposes (e.g., specimens geo-referenced to the country centroid) were removed from the dataset.

Finally, the expert botanists carrying out the Red List assessments verified all occurrences for each taxon, paying particular attention to spatial outliers that could indicate an error in a specimen’s identification or geo-referencing. Verification of taxonomic identification involved physically examining the herbarium specimens or at least online checking of an image scan where applicable. Not only did the experts detect (and rectify where possible) taxonomic and geographic errors, but they also identified unsuitable records, like those belonging to cultivated specimens or specimens that were locally extinct (e.g., due to habitat loss). Including such records in the calculation of the EOO or number of locations could result in an underestimation of extinction risk (Miller et al. 2012; Nic Lughadha et al. 2018). Records belonging to non-extant populations were flagged in the dataset (not removed) so they would not be used in calculating key geographic range parameters used in the Red List assessment process. Such information is valuable to the assessor to infer a continuing decline as applied in criterion B. Wrongly identified specimens and cultivated specimens were removed from the dataset.

## ﻿Results

The initial raw dataset contained data from 9956 specimens. The majority of these (83.4%) were deposited in the herbarium of Meise Botanic Garden, underlining its importance for the flora of Central Africa. Other herbaria represented in the dataset are B, BM, BRLU, C, COI, EA, EALA, EPU, FHO, GENT, H, HBG, IEC, IUK, K, KAW, KISA, LBV, LG, LISC, LISU, LSHI, LUKI, LWI, M, MA, MB, MHU, MO, MPU, NDO, NHR, NHT, P, PRE, SRGH, UPS, W, WAG, and YBI.

As part of the data enrichment, we transcribed locality data for 690 specimens, habitat data for 2802 specimens, and elevation data for 3796 specimens (this includes values indicating that information is ‘known to be unknown’). One-third of the specimens (33.1%) had no coordinates. During the ECAT project, 2923 specimens were geo-referenced, leaving 372 without spatial data. The new coordinates were derived mainly from maps and gazetteers; only for a small number of them (374) could they be copied from duplicates. After several quality checks on the geo-referencing, we adjusted the coordinates for 1774 specimens. For three-quarters of them, it concerned a relatively minor adjustment moving the occurrence up to 10 km. For the remaining quarter, this exceeded 10 km (up to as much as 3915 km). The taxonomic identification was updated for 509 specimens (changes due to synonymy or misspellings not taken into account). We removed 1046 specimens from the dataset on taxonomic or spatial grounds, leaving 8910 specimens in the cleaned dataset. After merging all duplicate specimens, we obtained a dataset with 6361 geo-referenced plant collection records.

### ﻿Taxonomic coverage

**Figure 1.**
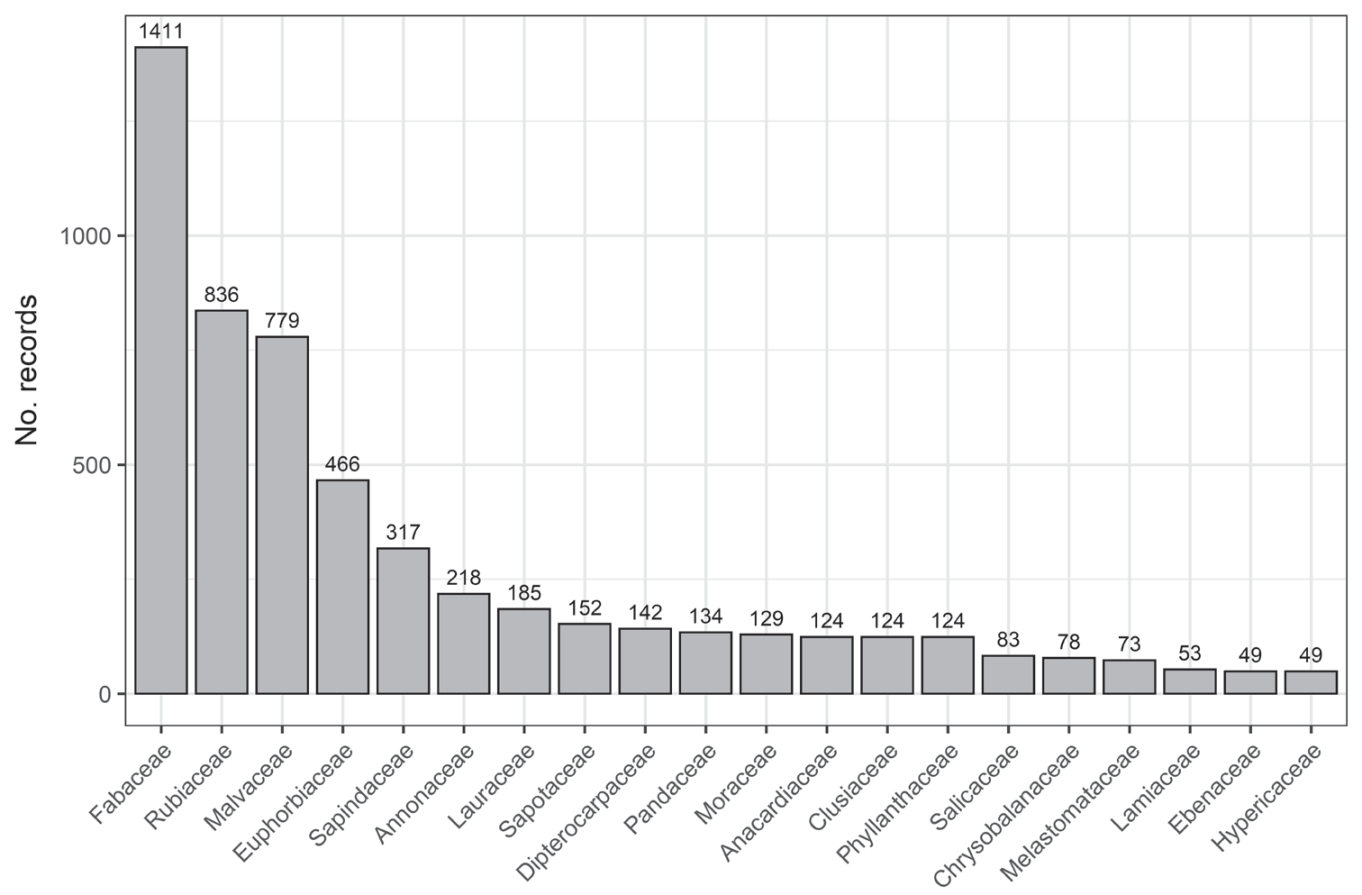
Taxonomic coverage: The top 20 families with the highest number of records of Central African endemic and sub-endemic trees in the ECAT dataset (totalling 6361 records and 337 taxa).

The ECAT dataset contains distribution data of 337 taxa at specific or infraspecific level (subspecies or variety) belonging to 153 genera in 52 families and 20 orders. The family classification follows APG IV (The Angiosperm Phylogeny Group 2016), except in some cases where the authors thought a more conservative approach to be appropriate (such as still recognising Flacourtiaceae, Sterculiaceae, and Tiliaceae). The difference in the number of taxa in the dataset (337) compared to our taxon list (347) arose because in the latter several species are present including their infraspecific taxa as separate entries, whereas the corresponding specimens in the dataset were all identified to infraspecific level. The number of records per taxon ranged between 1 and 130 (median: 10; mean: 19 records per taxon). The five most represented families are Fabaceae (22.2%), Rubiaceae (13.1%), Malvaceae (12.2%), Euphorbiaceae (7.3%), and Sapindaceae (5.0%) (Fig. 1).

### ﻿Taxonomic ranks

**Kingdom**: Plantae.

**Division**: Magnoliophyta.

**Class**: Magnoliopsida.

**Order**: Apiales, Arecales, Asterales, Boraginales, Brassicales, Celastrales, Ericales, Fabales, Gentianales, Geraniales, Lamiales, Laurales, Magnoliales, Malpighiales, Malvales, Myrtales, Proteales, Rosales, Santalales, Sapindales.

**Family**: Achariaceae, Anacardiaceae, Annonaceae, Apocynaceae, Araliaceae, Arecaceae, Asteraceae, Bignoniaceae, Boraginaceae, Burseraceae, Capparaceae, Celastraceae, Chrysobalanaceae, Clusiaceae, Combretaceae, Dichapetalaceae, Dipterocarpaceae, Ebenaceae, Euphorbiaceae, Fabaceae, Hypericaceae, Lamiaceae, Lauraceae, Linaceae, Malpighiaceae, Malvaceae, Melastomataceae, Meliaceae, Melianthaceae, Moraceae, Myrsinaceae, Myrtaceae, Octoknemaceae, Pandaceae, Pentaphylacaceae, Phyllanthaceae, Picrodendraceae, Pittosporaceae, Proteaceae, Putranjivaceae, Rhamnaceae, Rhizophoraceae, Rosaceae, Rubiaceae, Rutaceae, Salicaceae, Santalaceae, Sapindaceae, Sapotaceae, Scytopetalaceae, Thymelaeaceae, Violaceae.

**Common names**: flowering plants.

### ﻿Spatial coverage

#### General spatial coverage

**Figure 2.**
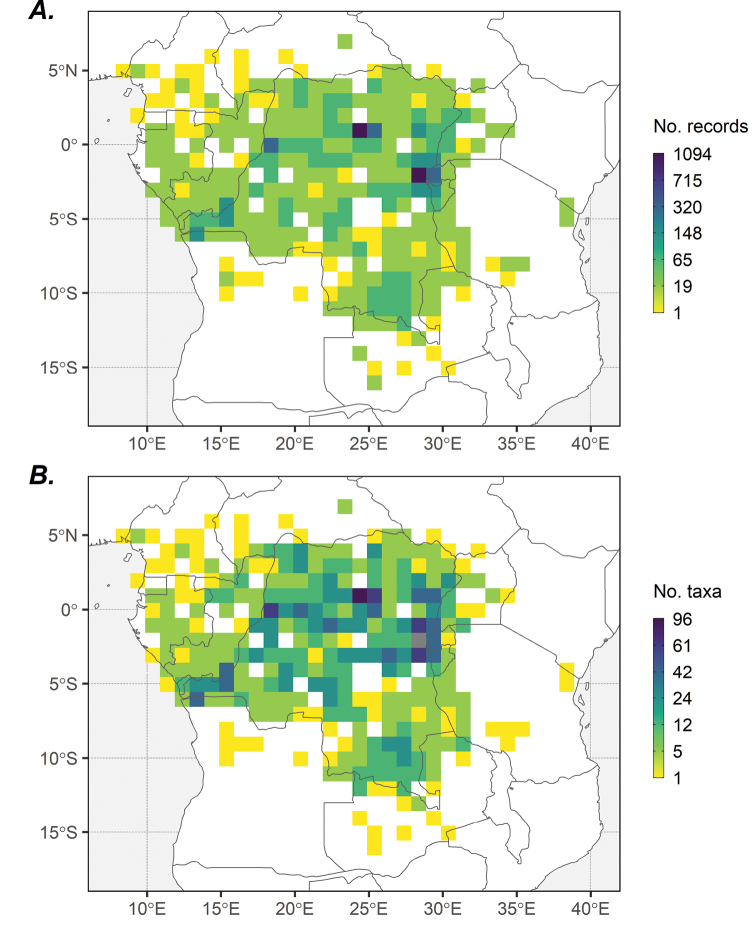
Spatial coverage: One-degree grid maps showing **A** number of records and **B** number of taxa of Central African endemic and sub-endemic trees in the ECAT dataset (totalling 6361 records and 337 taxa).

The occurrence data are relatively well distributed, albeit unevenly over the study area (Fig. 2A). Most records are from DR Congo (83.8%), followed by Rwanda (5.5%), Uganda (2.9%), and Burundi (2.4%), with the remaining records from outside Central Africa (Table 1). Collecting effort has been highest in the following areas: (1) the Greater Virunga Landscape, covering areas around the protected areas of Virunga National Park in DR Congo, Volcanoes National Park in Rwanda, and five national parks (NP) in Uganda (Bwindi Impenetrable NP, Mgahinga Gorilla NP, Queen Elizabeth NP, Rwenzori Mountains NP, and Semliki NP); (2) the Congo-Nile Divide of Rwanda and Burundi, to the east of the Albertine Rift, including Nyungwe NP and Kibira NP; (3) the region covering the eastern part (Kivu) of DR Congo; (4) the UNESCO Biosphere Reserve of Yangambi, situated in the north of DR Congo along the Congo River; (5) the UNESCO Biosphere Reserve of Luki, located in the south-west of DR Congo and about 120 km east of the Atlantic coast; (6) Kinshasa, the national capital of DR Congo; and (7) the area around Mbandaka, the capital of the Équateur province in DR Congo, located near the confluence of the Congo and Ruki Rivers. Logically, the patterns observed for the number of taxa in each grid cell (Fig. 2B) are rather similar since the two are highly correlated (Pearson’s r = 0.810).

**Table 1.** Spatial coverage: Number of specimens (total: 8910) and number of records (total: 3631) per country.

Country	No. specimens	No. records
Democratic Republic of the Congo	7750	5329
Rwanda	413	353
Uganda	186	184
Burundi	172	150
Republic of the Congo	143	123
Zambia	70	66
Gabon	44	41
The United Republic of Tanzania	37	37
Central African Republic	36	31
Cameroon	32	22
Angola	21	20
South Sudan	4	3
Equatorial Guinea (mainland)	2	2
**Total**	**8910**	**6361**

#### Coordinates

15°49'20"S to 06°31'00"N latitude; 08°48'00"E to 38°30'00"E longitude.

### ﻿Temporal coverage

**Figure 3.**
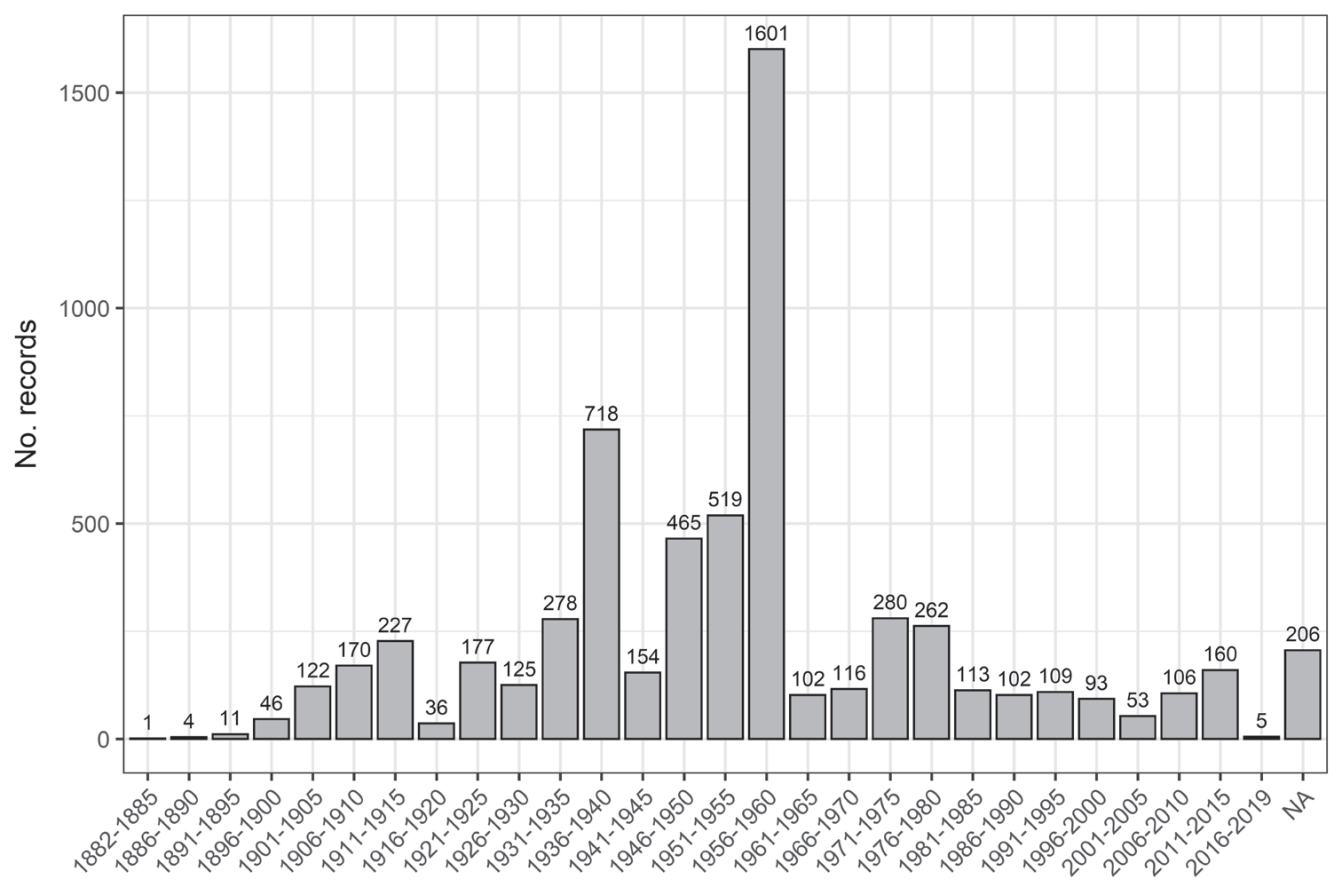
Temporal coverage: Number of records per 5-year periods from 1882 to 2019 of Central African endemic and sub-endemic trees in the ECAT dataset (totalling 6361 records and 337 taxa).

The ECAT dataset includes specimens collected between 1882 and 2019, with 206 records not having a date (Fig. 3). The periods of highest collecting activity were the 1930s and 1950s, with an exceptionally high level during the 1956–1960 period. There was reduced survey effort during the two World Wars and since the 1960s following the independence of DR Congo.

### ﻿Limitations of the data

For a tropical region to be considered reasonably well-known botanically (vascular plants), a rule of thumb is that the minimal level of botanical exploration should be at least 100 specimens per 100 km^2^ (Campbell and Hammond 1989). For Central Africa, this means that a minimum of 2.4 million herbarium specimens are needed, or by country, roughly about 2.35 million for DR Congo, 26,000 for Rwanda, and 28,000 for Burundi. The number of realised herbarium collections is estimated at 380,000 for DR Congo, 31,000 for Rwanda, and 37,000 for Burundi (Sosef et al. 2021). These numbers suggest that while Rwanda and Burundi can be regarded as “reasonably well known”, the botanical wealth of DR Congo remains “poorly known”. Moreover, the ECAT dataset suffers from the same sampling bias characteristic of many other natural history collections from tropical areas, with specimens collected mainly along roads, near urban populations, and in areas of specific botanical interest, with few collection records in remote areas (Fig. 2A). With respect to the temporal coverage, most specimens date back to the first half of the 20^th^ century and survey effort has decreased in the last few decades (Fig. 3). The gaps in both spatial and temporal coverage prompt the need for strategic and well-designed field surveys across the region, especially in remote and data-sparse areas with (relatively) intact vegetation and areas where threatened endemics occurred in the past but have not been surveyed for a long time. Such fieldwork is critical to assessing the status of extant populations under changing environmental conditions and identifying priority populations for immediate conservation. New, up-to-date occurrence records are pivotal for updating IUCN Red List assessments, especially for data deficient and threatened taxa, to make informed decisions regarding their conservation and management. Unfortunately, several factors hinder collection activities in Central Africa, including limited funding, inadequate infrastructure, and armed conflicts in DR Congo.

### ﻿Dataset description

**Object name**: Darwin Core Archive ECAT: Endemic and sub-endemic Central African Trees.

**Character encoding**: ISO-8859-1.

**Format name**: Darwin Core Archive format.

**Format version**: 1.5.

**Distribution**: https://zenodo.org/record/7007770.

**Publication date of data**: 2022-08-18.

**Licenses of use**: Creative Commons Attribution (CC-BY) 4.0 License.

**Metadata language**: English.

**Date of metadata creation**: 2022-08-18.

**Hierarchy level**: Dataset.

**Provided fields**: language, institutionCode, collectionCode, basisOfRecord, occurrenceID, catalogNumber, recordNumber, recordedBy, georeferenceVerificationStatus, occurrenceStatus, disposition, associatedReferences, otherCatalogNumbers, occurrenceRemarks, materialSampleID, eventDate, year, month, day, habitat, eventRemarks, continent, country, countryCode, stateProvince, locality, verbatimElevation, locationRemarks, decimalLatitude, decimalLongitude, geodeticDatum, coordinateUncertaintyInMeters, verbatimCoordinates, identificationRemarks, scientificName, kingdom, phylum, class, order, family, genus, specificEpithet, infraspecificEpithet, taxonRank, taxonRemarks.
